# The effects of nano-curcumin supplementation on adipokines levels in obese and overweight patients with migraine: a double blind clinical trial study

**DOI:** 10.1186/s13104-022-06074-4

**Published:** 2022-05-23

**Authors:** Mohsen Sedighiyan, Mina Abdolahi, Elham Jafari, Zahra Vahabi, Sara Sohrabi Athar, Shima Hadavi, Mahnaz Narimani Zamanabadi, Mir-Saeed Yekaninejad, Mahmoud Djalali

**Affiliations:** 1 Department of Cellular and Molecular Nutrition, School of Nutritional Sciences and Dietetics, Tehran University of Medical Sciences, Poursina Street, Tehran, Iran; 2 Amir Alam Hospital Complexes, Tehran University of Medical Sciences, Sa’adi Street, Tehran, Iran; 3 Headache Research Center, Sina Hospital, Tehran University of Medical Sciences, Imam Khomeini Street, Tehran, Iran; 4 Department of Geriatric Medicine, Ziaeian Hospital, Tehran University of Medical Sciences, Ghazvin street, Tehran, Iran; 5 Memory and Behavioral Neurology Division, Roozbeh Hospital, Tehran University of Medical Sciences, Kargar Street, Tehran, Iran; 6 Department of Human Nutrition, Faculty of Medicine, Urmia University of Medical Sciences, Resalat Street, Urmia, Iran; 7 Treatment Department, Tehran University of Medical Sciences, Hafez Street, Tehran, Iran; 8 Department of Anesthesiology, Faculty of Medicine, Tehran Medical Sciences, Islamic Azad University, Tehran, Iran; 9 Department of Epidemiology and Biostatistics, School of Public Health, Tehran University of Medical Sciences, Tehran, Iran

**Keywords:** Migraine, MCP-1, Resistin, Visfatin, Serum levels, Nano-curcumin

## Abstract

**Objective:**

The present study aimed to investigate the effects of nano-curcumin supplementation on adipokines levels and clinical signs in obese and overweight patients with migraine.

**Results:**

Forty-four patients with episodic migraine participated in this clinical trial and were divided into two groups nano-curcumin (80 mg/day) and the control group over 2-month period. At the baseline and the end of the research, the serum levels of MCP-1, Resistin, and Visfatin were measured using the ELISA method. In addition, the headache attack frequencies, severity, and duration of pain were recorded. The results of the present study showed that nano-curcumin can significantly reduce MCP-1 serum levels in the nano-curcumin supplemented group (P = 0.015, size effect = 13.4%). In the case of resistin and visfatin, nano-curcumin supplementation exerted no statistically significant changes in serum levels (P > 0.05). Nano-curcumin also significantly reduced the attack frequencies, severity, and duration of headaches (P < 0.05). These findings indicate that targeting curcumin can be a promising approach to migraine management. However, further comprehensive human trials are needed to confirm these findings.

**Trial Registration:**

This study was registered in the Iranian Registry of Clinical Trials (IRCT) with ID number: IRCT20160626028637N2 on the date 2020-07-10.

**Supplementary Information:**

The online version contains supplementary material available at 10.1186/s13104-022-06074-4.

## Introduction

Migraine is a disabling disorder characterized by recurrent attacks affecting the patient's quality of life [1]. Migraine is the third most common disease in the world and the prevalence of migraine in the general population is 12% [2, 3]. Clinical studies show obesity is an important risk factor for the progression and migraine attacks [4, 5] which is mainly related to inflammatory mediators and adipokines [6]. It has been demonstrated that Resistin, Visfatin, and Monocyte chemoattractant protein (MCP)-1 levels increase during migraine attacks and are associated with increased pain intensity [7–9]. These adipokines are involved in migraine pathogenesis as induce inflammation and pain mediators [7, 10, 11]. Resistin directly stimulates Nuclear Factor kappa B (NFκB) and increases Tumor Necrosis Factor (TNF)-α and Interleukin (IL)-6 [12]. Visfatin also stimulates the expression of inflammatory cytokines such as IL-1, IL-6, and TNF-α, leading to increased activity of NFκB [13]. Additionally, MCP-1 is associated with inflammatory mediators such as TNF-α. Its expression by glia cells in the central nervous system increases neurogenic inflammation, which in turn is associated with headache attacks [14].

Based on evidence some compounds can exert modulatory effects on adipokine, pain intensity, and inflammation such as curcumin [15, 16]. Curcumin, the yellow active ingredient of turmeric, is derived from curcumalonga rhizome [17]. The bioavailability of curcumin is very low but nanoparticles of curcumin (nano-curcumin) which was used in this study have a high absorption [18].

The curcumin carries out regulatory properties on resistin, visfatin, and MCP-1; Curcumin can reduce mRNA expression and visfatin protein levels and NFκB activity [19] and exert its MCP-1-lowering effects through down-regulating the mitogen-activated protein kinase (MAPK) and NF-κB pathway [20]. Additionally, curcumin in human endothelial cells activated by resistin inhibits the expression of pro-inflammatory molecules [21]. It is clear that migraine changes the pattern of adipokines, and even some medications used to migraine treatment reduce adipokines concentration such as visfatin [22, 23] indicating the important role of adipokines in the treatment of migraines. Although the regulatory effects of curcumin on adipokines have been demonstrated in cellular and animal studies, human studies are very limited. In this regard, the present study hypothesizes that nano-curcumin can exert modulatory effects on adipokines involved in migraine pathogenesis resulting in an improvement of the clinical signs of migraine. Thus, the present study aimed to investigate the effects of nano-curcumin supplementation on adipokines levels in obese and overweight patients with migraine.

## Main text

### Method and materials

#### Study design and patients

This research was conducted as a randomized clinical trial (RCT) study. Forty-four episodic migraine patients participated (42 females and 2 males) from April to September 2021 at the Tehran University of Medical Sciences (TUMS), Tehran, Iran. Inclusion criteria of this research were including age 20–50, body mass index (BMI) 25–35, any disorders such as cancer, diabetes, thyroid, liver or renal disease, cardiovascular disorders or inflammatory condition, no supplements consumption, special activity, and specific diet at least in last 3 months and diagnosis of episodic migraine based on International Headache Society criteria by the neurologist. Exclusion criteria included pregnancy during the trial, allergic reaction to curcumin, any alternation in routine treatment, consumption of less than 90% of supplements, and any inflammatory diseases or headache attacks leading to more than 2 weeks of use of analgesics.

At the baseline, written informed consent approved by the TUMS ethics committee was obtained from all participants. The goals, benefits, and potential risks of the present study were explained. The baseline data, medical history, medications, headache frequencies, headache duration based on participants’ reports were recorded at the first and end of the trial. The pain severity was recorded using visual analogue scale (VAS) measurement.

To determine compliance, patients were contacted per 2 weeks to ask about supplements consumption or side effects. Less than 90% consumption of the total supplements or placebo at the end of the study was defined as non-compliant. At the end of the study, the rate of supplementation in the nano-curcumin and control group was 96.4% and 94.2% respectively, so none of the patients were considered non-compliant or none of them lost more than 10% of the supplement. Generally, 1 patient in the control group withdrew the research due to unwillingness to cooperate. This study adheres to CONSORT guidelines.

#### Nano-curcumin supplementation

We used stratified randomization to control gender and BMI variables. The sample size was determined by the formula. The participants were divided into 2 groups by Permuted Block Randomization method. In each group 22 patients including 21 women and 1 man were entered included (I) the nano-curcumin group and (II) the control group. The participants in group 1 take 80 mg nano-curcumin including two capsules 40 mg/day and the control group received a nano-curcumin placebo containing oral paraffin oil, 2 capsules/day for 2 months. The nano-curcumin and placebo capsules were similar in color, size, and shape. In addition, the nano-curcumin or placebo capsules were coded by a third person.

#### Measurement of serum concentration of adipokines

At baseline and after 2-month intervention, 10 ml blood samples were taken from participants. The serum of patients was collected after10 minutes of centrifugation at 3000 RPM and stored at − 80 °C for subsequent measurements of resistin, visfatin, and MCP-1 levels by the enzyme-linked immunosorbent assay (ELISA). The ELISA method was conducted according to Kit protocols (Mediagnost, Germany) and read by an ELISA reader (ELx800 Absorbance Microplate, USA). The best standard curve and its equation were obtained using “Curve expert 1.4” software. The corrected optimal density (OD) (read OD- blank OD) was used to determine the diagrams and calculate concentrations. Then, the concentration of the adipokines in the samples was determined using the obtained equation (Additional file 1: Figure S1).

#### The clinical sign of headache recording

At the baseline and end of the trial, the headache frequencies (number of attacks per week) and headache attacks duration (average hours of headache) were recorded based on patient self-reports. The VAS measurement was used for pain severity recording at the start and end of the intervention. According to the VAS scale, the patients rated their pain (scoring 0 –10). VAS divides the pain severity into five categories as defined 0: No pain, 1–3: Mild pain, 4–6: Moderate pain, 7–9: Severe pain, and 10: Worst pain [24].

#### Statistical analysis

The data was transferred to SPSS software 22 for analysis. The normality of data was examined by the Kolmogorov–Smirnov distribution test. The Paired t-test and Independent t test were used for comparison of within and between groups of normal data respectively. Wilcoxon and Mann U Witney tests were used respectively for within and between-group comparison of data not normally distributed. Chi-squared test was used to compare qualitative variables. Analysis of covariance (ANCOVA) test was used to remove the effect of confounding variables. Data are expressed as mean ± Standard Error of the Mean (SEM). The P value ≤ 0.05 was accepted as a statistically significant difference. The intention to treat method was used to replace missing data.

### Results

#### Baseline participants information

The baseline and clinical information of patients in nano-curcumin and control groups are presented in Table 1. As shown, none of the baseline data showed significant differences between groups (Table 1). Also, no side effects were seen in any of the study groups.

**Table 1:** Baseline Participants information in nano-curcumin and control groups

Characteristics	Nano-curcumin group N = 22	Control group N = 22	P value
Anthropometric measurements			
Gender			
Female	21 (95.5%)	21 (95.5%)	0.75^a^
Male	1 (4.5%)	1 (4.5%)	
Age (years)	39.27 ± 2.15	41.00 ± 2.42	0.59^b^
Weight (kg)	77.52 ± 2.37	74.18 ± 1.66	0.25^b^
Height (cm)	161.82 ± 1.58	160.50 ± 1.53	0.55^b^
BMI (kg/m2)	29.66 ± 0.86	29.03 ± 0.75	0.40^b^
WC (cm)	98.18 ± 1.05	96.91 ± 1.08	0.58^b^
Clinical characteristics			
Onset of migraine age (year)	28.06 ± 2.53	26.14 ± 1.94	0.54^b^
Headache frequency (number/week)	3.59 ± 0.38	3.23 ± 0.37	0.43^c^
Duration of attacks (hours)	14.41 ± 2.45	17.55 ± 3.21	0.70^c^
Severity of pain (VAS scoring 0–10)	7.45 ± 0.38	7.64 ± 0.31	0.87^c^

All values are expressed as means ± SE or numbers

*BMI* Body Mass Index, *WC* Waist Circumference

^a^Chi-squared test

^b^Independent t test

^c^Mann U Witney Test

#### Adipokine serum levels

A significant reduction in the serum levels of MCP-1 was observed in patients who received nano-curcumin supplements which were significant between groups (P = 0.015, size effect = 13.4%) (Additional file 2: Figure S2). In the case of resistin and visfatin, no statistically significant reduction was found either within or between groups, even after adjustment based on baseline serum levels as a confounder (P = 0.60, size effect = 0.7% and P = 0.24, size effect = 3.4% respectively) (Table 2).

**Table 2:** Serum levels of adipokines

Adipokines	Nano-Curcumin group N = 22	Control Group N = 22	P Value^b^	Effect size
MCP-1 (ng/ml)				
Before	166.13 ± 10.94	168.47 ± 10.62	0.76	13.4%
After	153.97 ± 9.57	172.87 ± 11.12	0.015^c^	
Difference	− 12.15 ± 4.60	4.39 ± 2.07	0.009	
P value^a^	0.01	0.06		
Resistin (ng/ml)				
Before	124.89 ± 8.13	129.59 ± 8.47	0.7	0.7%
After	119.46 ± 6.48	126.24 ± 7.80	0.60^c^	
Difference	− 5.42 ± 7.43	− 3.34 ± 6.54	0.23	
P value^a^	0.49	0.58		
Visfatin (ng/ml)				
Before	19.82 ± 1.04	19.73 ± 0.90	0.91	3.4%
After	19.09 ± 1.00	19.57 ± 0.87	0.24^c^	
Difference	− 0.74 ± 0.40	− 0.15 ± 0.27	0.38	
P value^a^	0.21	0.55		

Data are reported as means ± SE

^a^Will Coxon t test

^b^Mann U Witney Test

^c^Analysis of Covariance (ANCOVA)

#### Clinical findings of headache

Based on Table 3, nano-curcumin significantly reduced headache attack frequencies (per week) between groups (P < 0.001, size effect = 47%). As well, a significant reduction was found in the duration of headache (hour) in the nano-curcumin-received group (P < 0.001) and this reduction also was statistically significant between groups (P < 0.001, size effect = 34%).

**Table 3:** Clinical sign of headache

Headache	Nano-curcumin group N = 22	Control group N = 22	P value^b^	Effect size
Frequencies (number/ week)				
Before	3.23 ± 0.37	3.59 ± 0.38	0.43	47%
After	1.82 ± 0.19	3.95 ± 0.31	< 0.001c	
Difference	− 1.40 ± 0.37	0.36 ± 0.22	< 0.001	
P value^a^	0.002	0.12		
Duration (hour)				
Before	17.55 ± 3.21	14.41 ± 2.45	0.7	34%
After	4.42 ± 0.87	12.34 ± 2.02	< 0.001c	
Difference	− 13.12 ± 3.08	− 2.06 ± 1.85	0.004	
P value a	< 0.001	0.42		
Severity (visual analogue scale (VAS) scoring)				
Before				
No pain	0 (0%)	0 (0%)	0.87	37%
Mild pain	0 (0%)	1 (4.5%)		
Moderate pain	8 (36.4%)	6 (27.3%)		
Sever pain	12 (54.5%)	13 (59.1%)		
Worst pain	2 (9.1%)	2 (9.1%)		
After				
No pain	1 (4.5%)	0 (0%)	< 0.001^c^	
Mild pain	6 (27.3%)	1 (4.5%)		
Moderate pain	13 (59.1%)	7 (31.8%)		
Severe pain	2 (9.1%)	13 (59.1%)		
Worst pain	0 (0%)	1 (4.5%)		
P value^a^	< 0.001	0.48		

Data are reported as means ± SE

^a^Will Coxon t test

^b^Mann U Witney Test

^c^Analysis of Covariance (ANCOVA)

In the case of pain severity, based on VAS measurement, the nano-curcumin supplementation significantly reduced headache severity (P < 0.001) with a size effect of 37%) (Table 3).

### Discussion

In the present trial, 44 patients with episodic migraine were enrolled and underwent 2- months nanocurcumin or placebo supplementation to evaluate the effects of nano-curcumin on resistin, visfatin, and MCP-1 levels and clinical manifestation of headache in migraine patients.

Adipokines play key roles in multiple physiological functions including weight regulation, endothelial performance, inflammation, and immune response [7]. These adipokines such as resistin, visfatin, and MCP-1 have abundant receptors in the central nervous system, are involved in migraine pathogenesis [10, 12, 14]. The resistin, visfatin, and MCP-1 concentration increase in migraine patients' serum or cerebrospinal fluid. Also, there is a significant correlation between these adipokines with clinical symptoms in migraine [7, 11, 13].

This research showed that nano-curcumin significantly reduced MCP-1 serum levels compared to the placebo group. Parallel with our finding Ganjali et al. showed in obese patients, curcumin significantly reduced MCP-1 serum levels [25]. In contrast, in some clinical studies, curcumin supplementation did not have significant effects on MCP-1 serum levels [26].

In the control group, the MCP-1 changes are close to significant levels (P = 0.06), but these changes are increased whereas in the nano-curcumin group decreased, their differences between groups are significant (P = 0.009). The regulatory effects of curcumin on MCP-1 occur through several mechanisms including downregulating of MCP-1 mRNA and receptor expression, suppressing MAPK and NF-κB signaling, and reducing MCP-1 activity/production by influencing AMP-kinase signaling [20, 27, 28]. But, in the placebo group, the MCP-1 level showed an increasing trend. According to evidence, the MCP-1 levels of migraine patients increase both in the attack and intervals of attack phase [8] which may be enhanced in the absence of inhibitory effects of curcumin in our control group. However, next studies with more participants are needed to confirm these results.

Additionally, nano-curcumin couldn’t reduce visfatin and resistin concentration significantly. Contrary to our results Islami et al. reported curcumin significantly reduced resistin level in obese participants [29, 30]. As well, experimental studies confirm significant regulatory effects of curcumin on visfatin levels [31]. Also, the inhibitory effects of curcumin on resistin-induced inflammation in human endothelial cells have been demonstrated [21]. Curcumin can significantly reduce visfatin mRNA expression and protein levels along with NF-κB activity. NF-κB is able to bind to the promoter of the visfatin gene and increase its expression [19].

In addition, nano-curcumin supplementation significantly reduced attack frequency, headache duration, and pain severity in participants. previously, Abdolahi et al. reported that nano-curcumin treatment decreased headache frequency and pain severity in migraine while did not exert a significant effect on headache duration [32–34]. Curcumin exerts its analgesic effects through the following mechanisms: downregulating of pain mediators (TNF-α, IL-6), calcitonin gene-related peptide, and substance P [35], suppressing nociceptor activity [36] and NF-κB signaling [37].

The number of females was higher in this study. Based on pieces of evidence, migraine is three times more common in women than in men attributed to factors including sex hormones, nutrition, economic-social status, and lifetime [38].

The present trial is the first study of the effects of nano-curcumin supplementation on adipokine levels and clinical signs of migraine. In this research, we found that nano-curcumin significantly reduced MCP-1 levels, attack frequencies, pain severity, and duration of headaches. Thus, targeting curcumin can be a promising approach to migraine management. However, to clarify the modulatory mechanisms of curcumin at mRNA expression and protein production levels, it is suggested to determine the gene expression and western blot of these adipokines in future studies, which was one of the limitations of the present trial.

## Limitations

There were several limitations in this study; the small sample size, short duration, and no measurement of adipokines mRNA or western blot. Additionally, the human studies on curcumin effects on headache and adipokines are very limited and the mechanisms proposed are often based on cellular and animal studies, which cannot be generalized to humans.

## Supplementary Information

**Additional file 1**: **Figure 1.** ELISA standard curve of MCP-1, Resistin and Visfatine

**Additional file 2:** **Figure 2.** MCP-1 serum levels in nano-curcumin and control groups. A significant reduction has been observed in MCP-1 levels in nano-curcumin group. This difference also is significant between groups. *P value <0.05. Each value represents mean ± SEM.

## Data Availability

“The datasets supporting this study results are included within the manuscript”. More information could be available by contact with this email address: m.sedighiyan86@gmail.com.

## Abbreviations

AMP: Adenosine monophosphate
BMI: Body mass index
IFNγ: Interferon gamma
IL: Interleukin
MAPK: Mitogen-Activated Protein Kinase
MCP-1: Monocyte chemoattractant protein-1
NF-κB: Nuclear Factor kappa B
TNF: Tumor Necrosis Factor
VAS: Visual Analogue Scale
