# Supplementary figures and images for: The effects of nano-curcumin supplementation on adipokines levels in obese and overweight patients with migraine: a double blind clinical trial study
Source: BMC Res Notes. 2022 May 23;15:189. doi: 10.1186/s13104-022-06074-4 (PMC9125853; doi:10.1186/s13104-022-06074-4)

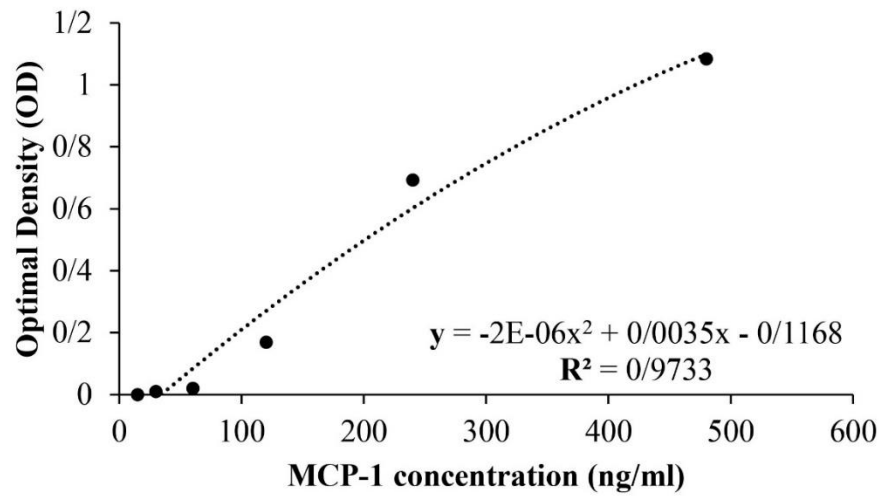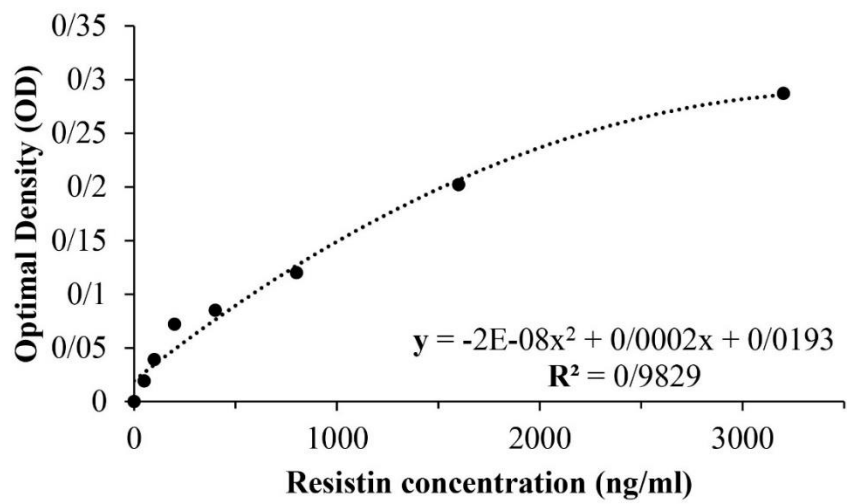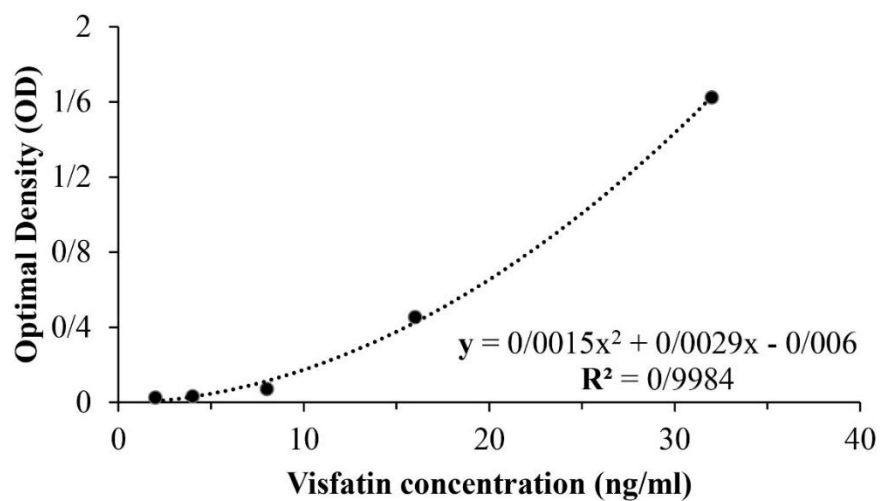

**Figure 1. ELISA standard curve of MCP-1, Resistin and Visfatine**

Supplement: Supplementary file 1 — Additional file 1: Figure 1. ELISA standard curve of MCP-1, Resistin and Visfatine [file 13104_2022_6074_MOESM1_ESM.pdf]
