# Supplementary material for: The effects of nano-curcumin supplementation on adipokines levels in obese and overweight patients with migraine: a double blind clinical trial study
Source: BMC Res Notes. 2022 May 23;15:189. doi: 10.1186/s13104-022-06074-4 (PMC9125853; doi:10.1186/s13104-022-06074-4)
Supplement: Supplementary file 2 — Additional file 2: Figure 2. MCP-1 serum levels in nano-curcumin and control groups. A significant reduction has been observed in MCP-1 levels in nano-curcumin group. This difference also is significant between groups. *P value <0.05. Each value represents mean ± SEM. [file 13104_2022_6074_MOESM2_ESM.pdf]

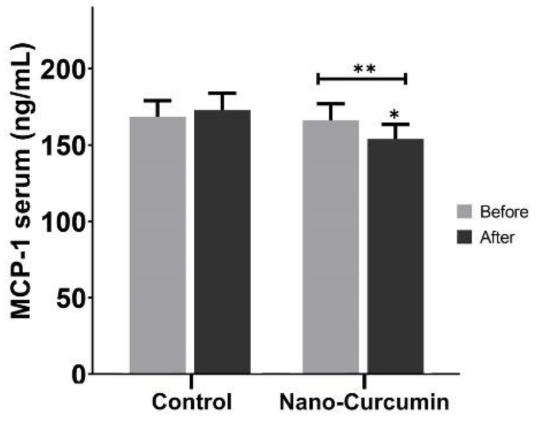

Figure 2. MCP-1 serum levels in nano-curcumin and control groups. A significant reduction has been observed in MCP-1 levels in nano-curcumin group. This difference also is significant between groups. * P value <0.05. Each value represents mean ± SEM.
